# Characterization of the complete chloroplast genome of endangered Cycads *Zamia fischeri* Miq. ex Lem

**DOI:** 10.1080/23802359.2018.1508387

**Published:** 2018-10-26

**Authors:** Jin-Yan Lei, Damien Daniel Hinsinger, Guo-Feng Jiang

**Affiliations:** a Plant Ecophysiology & Evolution Group, Guangxi Key Laboratory of Forest Ecology and Conservation, College of Forestry, Guangxi University, Nanning, Guangxi, PR China;; b State Key Laboratory for Conservation and Utilization of Subtropical Agro-bioresources, Guangxi University, Nanning, Guangxi, PR China

**Keywords:** *Zamia fischeri*, chloroplast, evolution, Cycads

## Abstract

The whole chloroplast (cp) genome sequence of *Zamia fischeri* has been characterized. The cp genome length was 164,767 bp in length, with a GC content of 39.7%, containing a large single copy (LSC) of 90,226 bp, a small single copy (SSC) of 23,223 bp, and a pair of inverted repeats (IRs) of 25,659 bp. The genome contained 127 genes, including 88 protein-coding genes, 31 tRNA genes, and 8 rRNA genes. A phylogenetic analysis based on complete chloroplast genomes in Cycads indicates that *Z. fischeri* clustered with another *Zamia* (*Z. furfuracea).* This complete chloroplast sequence offers a promising tool for further species identification, population genetic conservation, and evolutionary studies of Zamiaceae, as well as for Cycadales.

Cycads are iconic relict species (Brenner et al. 2003), despite a recent diversification (Nagalingum et al. 2011; Xiao and Möller 2015; Jiang et al. 2016). With a total of 10 accepted genera and 351 accepted species in three families (Cycadaceae, Strangeriaceae, and Zamiaceae), Cycads are found in most of the tropical and subtropical regions (Calonje et al. 2013–2018). In the family Zamiaceae, *Zamia* consists of 77 species, most being endangered at different levels (Vovides and Chemnick 2010; Calonje et al. 2013–2018). *Zamia fischeri* Miq. is endemic to Mexico (San Luis Potosí, Querétaro, and Tamaulipas states) and is found from arid areas and open habitats to rainforests. It is listed as an endangered species due to severe natural habitat destruction (Vovides and Chemnick 2010). Therefore, a coordinated effort is urgently required to ensure its conservation, either *in situ* or *ex situ*.

Plastomes (cpDNA) used in conservation studies have been demonstrated to provide useful and abundant information on genetic diversity and evolution in many taxa (Ye et al. 2014; Gao and Gao 2017), and showed especially high-resolution phylogenetic tree in Cycads (Jiang et al. 2016). In this study, we assembled and characterized the plastome sequence of *Z. fischeri* based on Illumina pair-end data, and built a phylogenetic tree using plastomes available in Cycads.

Leaves from an individual *Z. fischeri* were collected in Xishuangbanna Tropical Botanical Garden (Menglun, PR China, 21°55′N,101°15′E). Total genomic DNA was extracted as previously described (Jiang et al. 2016; Xu et al. 2017). Library construction and sequencing were processed by Novogene (Beijing, PR China) using an Illumina HiSeq X Ten system (Illumina, San Diego, CA), according to the manufacturer instructions. We performed a *de novo* assembly as described previously (Hinsinger and Strijk 2017; Jiang et al. 2016). Genome annotation was performed using CpGAVAS (Liu et al. 2012).

We reconstructed the 164,767 bp long chloroplast of *Z. fischeri* (GenBank accession number MH311043). It contained a LSC, SSC, and a pair of inverted repeats (IRa and IRb) of 90,226, 23,223, and 25,659 bp, respectively (Figure 1). We identified 127 genes, including 88 protein-coding genes, 31 tRNA genes, and 8 ribosomal RNA genes. Of these genes, 13 genes were duplicated in the IR regions, including 4 protein-coding genes (*ndhB, ycf2, rps7, rps12*), 5 tRNA genes (*trnH-GTG, trnL-CAA, trnN-GTT, trnR-ACG*, trnV-GAC), and 4 rRNA genes (*4.5S, 5S, 16S, 23S*). Five genes (*ndhA, ndhB, rpl2, rpoC1, rps12*) contained one intron while two genes contained two introns (*clpP, ycf3*). The overall GC content of the plastome of *Z. fischeri* was 39.7%, while the GC content in LSC, SSC, IRa, and IRb regions were 38.8%, 36.9%, 42.4%, respectively.

**Figure 1.**
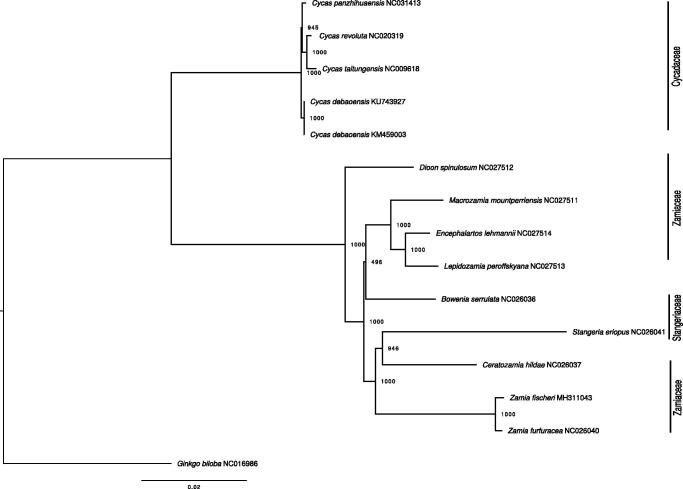
ML phylogenetic tree of the 14 available chloroplasts of Cycadales retrieved from GenBank, plus the plastome of *Ginkgo biloba* as outgroup. Bootstraps values (1000 replicates) are shown at the nodes. Scale in substitution per site.

Fourteen plastomes of cycads were retrieved from GenBank (accessed 2018/05/15), plus *Ginkgo biloba* as an outgroup (Figure 1), and aligned with MAFFT (Katoh and Standley 2013). We built a maximum likelihood (ML, TPM1uf + I + G model, 1000 bootstraps) tree using PHYML v3.3 (Guindon et al. 2009). All but one nodes were highly supported (BP ≥94%), with the two *Zamia* clustering together. The results of this phylogenetic analysis are highly consistent with a previous plastome-based study (Jiang et al. 2016). The plastome of *Z. fischeri* provides a useful bio-resource that will help to assess population diversity and demography for conservation purposes, and will also benefit to further genetic studies in Zamiaceae.
